# Eye Diseases and Impaired Vision as Possible Risk Factors for Recurrent Falls in the Aged: A Systematic Review

**DOI:** 10.1155/2012/271481

**Published:** 2012-08-15

**Authors:** Liisa Salonen, Sirkka-Liisa Kivelä

**Affiliations:** ^1^Department of Family Medicine, University of Turku, Lemminkäisenkatu 1, 20014 Turku, Finland; ^2^The Health Care Center of Parainen, Vapparintie 15 A, 21600 Parainen, Finland; ^3^Unit of Family Medicine, Turku University Hospital, PL 52, 20521 Turku, Finland; ^4^Satakunta Hospital District, Sairaalantie 3, 28500 Pori, Finland; ^5^Division on Social Pharmacy, University of Helsinki, Viikinkaari 9, 00014 University of Helsinki, Finland

## Abstract

*Background*. Recurrent falls are common among the aged. Vision is needed in maintaining balance, and impaired vision may be an intrinsic risk factor of recurrent falls. The aim was to perform a systematic review about the relationships between eye diseases or impaired vision and the risk of recurrent falls in the aged. *Material and Methods*. MEDLINE and CINAHL databases were searched in order to find longitudinal epidemiological studies about the associations between eye diseases or impaired vision and the risk of recurrent falls. Altogether 19 studies were found. A qualitative systematic analysis of these studies was performed. *Results and Conclusions*. The evidence about poor depth perception/stereoacuity and poor low-contrast visual acuity as risk factors of recurrent falls is quite convincing. Discrepant vision, a decrease in visual acuity, and loss of visual field may be risk factors, but more studies are needed. The results concerning the relationships between poor visual acuity and poor contrast sensitivity and the risk of recurrent falls are controversial. More studies about the relationships between different measures of vision and the risk of recurrent falls are needed before final conclusions about poor vision as a risk factor for recurrent falling can be done.

## 1. Introduction

Falls are common among the aged. One-third of community-dwelling people over the age of 65 years fall at least once a year [1–5]. The aged living in long-term institutions or in sheltered housing experience more falls than the home-dwelling aged [4]. Falls cause remarkable costs to the health care, and they may lead to long-term disabilities in the aged. Roughly 40% of serious falls lead to hospital admission, and 30–40% of the fallers admitted to hospitals are later transferred to nursing homes [6]. There are many reasons to develop prevention of falls. 

Falls may be classified in several ways. A common classification categorizes falls into three groups: falls that result from interference with the base of support (trips, slips), falls which result from externally applied push or self-induced displacement during bending, reaching, turning, or transfer, and falls which result from a physiological event that disrupts posture control mechanisms. Falls belonging to the first and second categories are usually accidental ones, and the person does not fall recurrently. Falls resulting from a physiological event are usually recurrent ones: the person falls several times a year [7]. 

Visual functioning, the ability to detect surroundings, is needed for posture control. Impaired vision may, thus, be a risk factor for falls, especially for recurrent falls. In preventing recurrent falls we need to know the specific features of vision that are risk factors for the recurrence. The development of recurrent falls prevention strategies should be based on the use of practical and exact tests of these risk factors. We decided to perform a systematic review about the relationships between eye diseases or impaired vision and the risk of recurrent falls in order to find the specific features of vision which increase the risk for falling recurrently.

## 2. Material and Methods

### 2.1. Search Strategy and Selection Criteria

Updated MEDLINE (1980–2/2008) and CINAHL (1980–2/2008) databases were searched from the Ovid database on February 20th, 2008 by using the strategy shown in Table 1. A second search in the MEDLINE and CINAHL databases was run on the 6th of May, 2010. The search strategy to MEDLINE was exactly the same as two years before, but the publication interval was altered to be 1980–2010. Because the Ovid database did not exist anymore, CINAHL was searched by a comparable strategy shown in Table 1. The third search to both databases was done on the 27th of May, 2012 with the strategies used in 2010. The only change done was that the publication interval was changed to be 5/2010–5/2012.

The language was restricted, and only English articles were taken into account. From the first search, critical reviews and longitudinal and cross-sectional studies were accepted though only retrospective or prospective longitudinal studies were included in this review. From the second and third searches, only retrospective or prospective longitudinal studies were accepted. 

**Figure 1:**
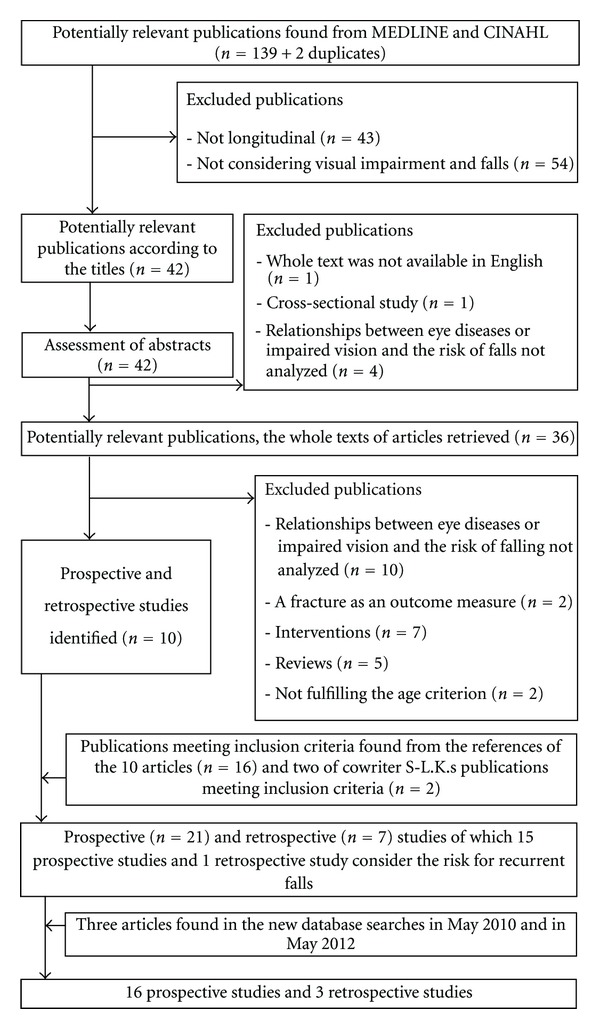
Flow chart.

A total of 141 citations were identified from the first search in MEDLINE and CINAHL (Figure 1). 141 citations included 2 articles twice. Both writers read through the titles.

 According to their titles, 42 articles considered the relationships between eye diseases or impaired vision and the risk of falls. 43 articles were excluded because there were not retrospective or prospective longitudinal studies or critical reviews. 54 articles were excluded because they did not consider impaired vision/eye diseases or falling. Abstracts of chosen 42 articles were read by one author (L. M. Salonen), who selected prospective and retrospective studies and critical reviews about relationships between eye diseases or impaired vision and the risk of falls to be read. Altogether six articles were excluded in this phase because they were cross-sectional studies (*n* = 1) or did not consider relationships between eye diseases or impaired vision and the risk of falling (*n* = 4) or were not published in English (*n* = 1). 

The whole texts of the remaining 36 articles were read by the same author (L. M. Salonen). In this phase, 24 articles were excluded because they were reviews (*n* = 5) considered interventions (*n* = 7) did not analyze relationships between eye diseases or impaired vision and the risk of falling (*n* = 10), or used a fracture as an outcome measure (*n* = 2). Only studies with mean age of the population over 65 years, or with the youngest participants older than 60 years if the mean age was not mentioned, were included. Two studies failed to meet the age criterion.

The reference lists of 10 original studies identified in this phase were checked by both authors, and additional 16 original studies meeting the above inclusion criteria were found and included in the material. In addition, two studies performed by one of the authors (S. L. Kivelä) of this paper were included in the material because they met the inclusion criteria, although they were not identified in the search. 

These 28 studies were classified according to their outcome variables into those considering recurrent falls (*n* = 16) and those considering nonrecurrent falls (*n* = 12). 

157 titles were achieved from MEDLINE and 72 from CINAHL in the second search run. Both writers of this paper read the abstracts to find additional articles considering impaired vision as a risk factor for recurrent falls. Only one new report was found. On the third search run, 35 titles were found from MEDLINE and 10 titles from CINAHL. Abstracts of all articles were read by both writers. Based on abstracts, 13 articles were possibly suitable to this literature review and the whole texts of these articles were read by L. M. Salonen. Two new articles fulfilled the inclusion criteria. 

The studies about the relationships between eye diseases or impaired vision and the risk of recurrent falls formed the material of this systematic review. The final material consisted of 16 prospective studies and 3 retrospective studies. 

The studies were classified according to the design (prospective/retrospective), measure of vision (objective near visual acuity, objective distant visual acuity, low contrast visual acuity, high-contrast visual acuity, distant contrast sensitivity, near contrast sensitivity, stereoacuity, visual field, perception of verticality, discrepant vision, subjective visual acuity, self-reported vision worsening, and eye diseases: glaucoma, cataract, and retinal diseases) used as a potential risk factor, and material (community-dwelling population, unselected population, intermediate care residents, and institutionalized population).

## 3. Results

### 3.1. Prospective Studies

#### 3.1.1. Unselected Populations

The potential risk factors taken into account in one report [8] (Table 2) considering an unselected population consisting of both home-dwelling and institutionalized participants were low contrast sensitivity and subjective poor vision. The results were adjusted for age, gender, and health variables.

Low contrast sensitivity was significantly related to the risk of recurrent falls, but subjective poor vision was not. 

#### 3.1.2. Community-Dwelling Populations

Ten prospective reports were found about relationships between impaired vision or eye diseases and the risk of recurrent falls in community-dwelling populations [3, 5, 9–16] (Table 2). Two reports concerned the same cohort of LASA [5, 14], and two reports concerned The Study of Osteoporotic Fractures in which all participants were women [10, 11]. 

Five reports considered poor visual acuity as a potential risk factor for recurrent falls [3, 9, 10, 13, 16]. In addition, in the LASA reports, subjective poor visual acuity (determined by asking if participants were capable of recognizing faces from 4 meters distance) was considered as a potential risk factor [5, 14]. Other potential risk factors considered were low contrast visual acuity [3, 13], change in visual acuity [11], poor distant contrast sensitivity [3, 9, 10, 13, 16], poor near contrast sensitivity [13], poor depth perception/stereoacuity [13, 16], visual field loss [10, 12, 13, 16], discrepant vision [9], and eye diseases such as glaucoma, cataract, or retinal diseases [11, 15].

In eight reports [5, 10–16], risk ratios were calculated and in five of these studies [10–13, 16] they were adjusted for several confounders. An analysis of variance with adjustment of age was used in two studies [3, 9].

Poor visual acuity was related to the risk of falling recurrently in one [9] of five studies. Subjective poor vision [5, 14] and reduced low contrast visual acuity [3, 13] were found to be risk factors in both studies in which they were measured and a reduction in visual acuity was related to the risk in the study using this criterion [11].

Poor stereoacuity and poor depth perception were detected to be significant risk factors in both studies in which they were measured [13, 16]. Visual field loss was related to the risk of recurrent falls in two reports [10, 12] out of four, and poor contrast sensitivity was related to the risk of recurrent falls in three reports [3, 9, 13] out of five. Near contrast sensitivity was measured separately in one study, and it was not related to the risk of recurrent falls [13]. One [15] out of two reports concerning eye diseases as potential risk factors found a positive association between eye diseases and recurrent falling. Discrepant vision was a significant risk factor for recurrent falls [9].

#### 3.1.3. Residents in Intermediate Care

The search produced four studies (Table 2) about relationships between impaired vision or eye diseases and the risk of recurrent falls among the residents in intermediate care: two studies in a hostel for the aged in Australia [17, 18], one study in intermediate care facilities in the USA [19], and one study in homes and apartments for the aged in The Netherlands [20]. 

The potential risk factors measured in these studies were decreased visual acuity [17–19], poor self-reported visual acuity [20], poor contrast sensitivity [18], visual field loss [17], and eye diseases [17]. Risk ratios were adjusted for age and sex in one study [20] and unadjusted in two studies [17, 19], and an analysis of variance adjusted for age was performed in one study [18].

Poor visual acuity was related to the risk for recurrent falling in two [17, 19] out of three studies and reduced contrast sensitivity was a significant risk factor in the only report using this measure [18]. Poor self-reported visual acuity, visual field loss, and eye diseases were not related to the risk of falling recurrently [17, 20].

#### 3.1.4. Institutionalized Populations

The results adjusted for confounders by the logistic regression analysis in the study in the aged in long-term institutional care [21] showed that the self-reported diagnosis of any eye disease was independently related to the risk of recurrent falls (Table 2).

### 3.2. Retrospective Studies

#### 3.2.1. Community-Dwelling Populations

3 retrospective studies [22–24] (Table 2) analyzed relationships between impaired vision or eye diseases and the risk of recurrent falls in a community-dwelling population. The potential risk factors measured in these studies were reduced visual acuity [22, 23], poor subjective visual acuity [22, 24], poor contrast sensitivity [22], loss of visual field [22], and eye diseases [22]. The results adjusted for age, gender and potential health variables showed reduced visual acuity [22, 23], poor contrast sensitivity [22], and loss of visual field [22] to be related to the risk of recurrent falls. Posterior subcapsular cataract was related to the risk, but other types of cataract, glaucoma, age-related macular regeneration, diabetic retinopathy, and subjective impaired visual acuity were not related to the risk [22, 24].

## 4. Discussion

Relationships between impaired vision or eye diseases and the risk of recurrent falls among the aged have been studied in a fairly large number of prospective and retrospective studies. Some studies have been done in unselected or community-dwelling populations, but the populations in some studies are selected, for example, the aged living in intermediate care facilities. Unselected populations are the most valuable materials for epidemiologic studies. For the qualitative analysis of this systematic review, the studies were divided into subgroups according to their materials, because differences in the selectivity of populations cause problems in the interpretation of the results.

The majority (*n* = 16) of the reports were prospective ones, and three studies used retrospective design. The studies using retrospective design were taken into account, although conclusions from their results must be done more critically than those from the results of prospective ones. By using a retrospective design, it is difficult to determine if certain identified risk factors such as poor functional abilities are consequences of previous falls. However, this is not a major problem when concentrating on impaired vision as a potential risk factor, because falls seldom result in visual impairment.

Other variables previously found to be related to the risk of recurrent falls were adjusted in a number of studies. The results of the studies which did not take into account these confounding variables are less valuable than the ones in which multivariate analyses were performed.

Registration of falls varied between the studies causing problems in assessing the reliability of the results and comparing the results with each other. A prospective follow-up with a fall record form is regarded as the most reliable method. Participants filled in fall record forms or reported falls regularly either by making written notes or by telephone in 13 prospective studies. Registration of falls by asking retrospectively is quite unreliable. Cummings et al. [25] studied 304 ambulatory persons in a prospective study and noticed that when asking participants one year after the baseline examination if they had fallen at least once during the previous year, 13% of fallers did not remember a fall event. If participants were asked one year after the baseline examination about falls during most recent 3 months, 32% of fallers denied falling. The proposed explanation is that participants remembered the baseline examination and therefore they could recall if a fall had happened before or after the examination. 

The materials of a majority (*n* = 7) of prospective studies and the materials of two retrospective studies performed in unselected or community-dwelling populations include some thousands of participants. In five studies done in unselected or community-dwelling populations the number of participants was less than one thousand. The follow-up periods in studies done in unselected or community-dwelling populations lasted at least one year. It seems likely that even weaker risk factors were detected in these studies. 

The numbers of participants in studies performed in intermediate care or in long-term institutions were quite small (*n* = 79 − 354). The follow-up periods lasted only three months or seven months in two studies, and three studies used a follow-up of one year or two years. We suggest that only stronger risk factors were detected among these selected populations. 

The studies differed in methods which were used to assess visual acuity and other specific features of vision. These differences caused problems in comparing the results and in drawing conclusions. Objective methods were used in 11 prospective studies, and 5 prospective studies were based on only subjective experiences of poor vision or on self-reported diagnosis of an eye disease. Two retrospective studies utilized objective measures and one was based on subjective experience of poor vision. Self-reported eye disease diagnoses are not very reliable measures. The methods to measure visual acuity differed between the studies. Binocular visual acuity, which is a more relevant measure than monocular visual acuity, was measured in most of the studies. Objective measurements are usually done in standardized conditions (e.g., lighting), which differ from daily living conditions. Subjective assessments are based on persons' experiences in their normal living surroundings. Therefore, a subjective assessment of vision may be quite valuable and informative. However, the question about subjective vision can be understood in a different way by different participants.

Five prospective studies done in unselected or community-dwelling populations with the adjustment of multiple confounders (more than gender or age) were found [8, 10–12, 16]. Both depth perception [16] and change in visual acuity [11] were measured in one study in which they proved to be significant risk factors. Visual field loss was a significant risk factor in two [10, 12] out of three studies. Poor contrast sensitivity was related to the risk of recurrent falls in one [8] out of three studies. Subjective poor vision [8] and self-reported eye diseases [11] were not found to be risk factors in the study in which they were measured. 

Three prospective studies done in community-dwelling populations adjusting results only for age [3, 9, 13] were found. Two studies [3, 13] found reduced low contrast visual acuity, and all three studies found reduced contrast sensitivity to be risk factors for recurrent falling. Streoacuity was a significant risk factor in the study in which it was measured [13]. 

Three prospective studies [5, 14, 15] done in community-dwelling populations without an adjustment of results found subjective poor vision to be related to the risk of recurrent falls. Self-reported diagnosis of an eye disease was a significant risk factor according to one study [15]. 

Two retrospective studies [22, 23] done in a community-dwelling populations with results adjusted for several confounders found poor visual acuity to be related to the risk of recurrent falls. Poor contrast sensitivity, loss of visual field, and posterior subcapsular cataract were risk factors to recurrent falls in one study [22].

One prospective study done in an institutionalized population found a self-reported eye disease to be a significant risk factor for recurrent falls after adjustment for several confounders [21]. Two prospective studies [18, 20] done in intermediate care used an adjustment only for age and/or sex and only reduced contrast sensitivity was related to risk of falling in one of these studies. Two prospective studies [17, 19] done in intermediate care without adjustment of the results found poor visual acuity to be a risk factor for recurrent falling.

## 5. Conclusions

The evidence about poor depth perception/stereoacuity and poor low contrast visual acuity as risk factors for recurrent falls is quite strong. Discrepant vision, a decrease in visual acuity within a relatively short time and loss of visual field may be risk factors, but more studies are needed. The results about the relationships between poor visual acuity and poor contrast sensitivity and the risk of recurrent falls are controversial.

More studies about the relationships between different measures of vision and the risk of recurrent falls are needed, because the results of the studies reviewed in this paper are partly controversial. Measures of functional vision which are easily determined in primary health care should be developed, and these measures should be included in these studies. The available results suggest that the measurement of vision should be included in prevention of future falls among the aged who have sustained an injurious fall and in health promotion programs for the aged.

## Figures and Tables

**Table 1:** Search strategies.

Search identification number	Search terms
Search strategy of the first search	

S1	Vision/or exp eye diseases/or exp vision disorders/
S2	((visual$ or vision or sight or eyesight or eye$1 or ocular) adj (impairment$ or disorder$ or disease$ or deficit$ or problem$ or disturb$ or lower$ or low or loss or reduc$ or decreas$ or weak$ or decay$ or diminish$ or fail$ or handicap$ or hindrance$ or damage$ or injur$)). tw.
S3	S1 or S2
S4	Accidental falls/or (falling$ or fall$1). ti.
S5	S3 and S4
S6	Limit 5 to (“aged (80 and over)” or aged <65 to 79 years> or “aged <80 and over>” or all aged (65 and over)”)
S7	Aging/or exp Aged/or (aging or ageing or elder$ or geriatr$ or gerontol$ or aged).tw.
S8	S5 and S7
S9	S6 or S8
S10	Remove duplicates from S9
S11	(predict$ or hazard$ or risk$ or progno$ or recurrent$ or repeat$ or repetit$ or frequen$ or continu$ or iterative). mp.
S12	S10 and S11
S13	Limit S12 to abstracts
S14	Limit S13 to English
S15	Limit S14 to yr = “1980–2008”

Search strategy of the second and third searches in CINAHL	

S1	(MH “Vision+”) or (MH “Vision Disorders+”)
S2	(Visual^∗^ or vision or sight or eyesight or eye or eyes or ocular) and (impairment^∗^ or disorder^∗^ or disease^∗^ or deficit^∗^ or problem^∗^ or disturb^∗^ or low or loss or reduc^∗^ or decreas^∗^ or weak^∗^ or decay^∗^ or diminish^∗^ or fail^∗^ or handicap^∗^ or hindrance^∗^ or damage^∗^ or injur^∗^)
S3	S1 or S2
S4	(MH “Accidental Falls”) or falling^∗^ or fall or falls
S5	S3 and S4
S6	S3 and S4
	Search options: Limiters-Age Groups: Aged: 65+ years
S7	MH “Aging+” or MH “Aged+” or (aging or ageing or elder^∗^ or geriatr^∗^ or gerontol^∗^ or senior^∗^)
S8	S5 and S7
S9	S6 or S8
S10	S6 or S8
	Search options: Limiters-Abstract Available; Publication Year from: 1980–2010 (the second search), 5/2010–5/2012 (the third search); English Language: Exclude MEDLINE records

**Table 2:** Studies about relationships between eye diseases or impaired vision and the risk for recurrent falls.

Authors, year	*N*	Mean age and range (yrs)	Registration of falls	Follow-up period	Results
Prospective studies					

Unselected populations					
De Boer et al., 2004 [8]	*n* = 1418 Females: 52%	Recurrent fallers: 77.3 ± 6.9 Nonrecurrent fallers: 75.1 ± 6.4 Range: 65−	Falls reported weekly on a calendar posted every 3 mo. Alternatively reported by telephone.	3 yrs	Integrated contrast sensitivity (HR = 1.53, 95% CI = 1.03–2.29) and low-frequency contrast sensitivity (HR = 1.66, 95% CI = 1.11–2.48) risk factors for recurrent falling after adjustment for confounders. Subjective visual acuity impairment not a risk factor

Community-dwelling populations					
Knudtson et al., 2009 [9]	*n* = 2256 Females: 58%	Nonrecurrent fallers: 71.3 ± 8.7, recurrent fallers: 76.0 ± 10.2 Range: 54–95	Questions about falling during past 12 mo made 5 years after ophthalmic examination,	1 yr	Poor best-corrected monocular visual acuity, poor contrast sensitivity, and discrepant vision associated statistically significantly with 2 or more falls (*P* > 0.05) after controlling for age

Coleman et al., 2007 [10]	*n* = 4216 Females: 100% Completed followup: *n* = 4071	79.9 ± 4.0 70−	By postcard or telephone every 4 mo.	1 yr	Severe binocular visual field loss associated with recurrent falls after adjusting for age, study site, and cognitive function (OR = 1.50, 95% CI = 1.11–2.02). No association between contrast sensitivity or visual acuity and recurrent falls when adjusted for age, study site, and cognitive function

Coleman et al., 2004 [11]	*n* = 2002 Females: 100%	76.4 ± 4.8 70−	Postcard or interview by telephone every 4 mo.	11.86 ± 1.25 mo	Declining visual acuity a risk factor for frequent falling. ORs after adjustment for baseline visual acuity and other confounders 2.08 (95% CI = 1.39–3.12) for loss of 1 to 5 letters using Bailey-Lovie chart, 1.85 (95% CI = 1.16−2.95) for loss of 6−10 letters, 2.51 (95% CI = 1.39–4.52) for loss of 11–15 letters, and 2.08 (95% CI = 1.01–4.30) for loss of >15 letters. Cataract, glaucoma, and retinal diseases not risk factors for recurrent falls

Ramrattan et al., 2001 [12]	At baseline: *n* = 6250 Females: 58% Completed follow-up: *n* = 5186	68 55−	Questions: “Did you fall >4 times in the past 2 years?” Asked 3 yrs after ophthalmic examination	2 yrs	Unilateral and bilateral visual field losses (VFLs) associated with a 6-fold risk of recurrent falls. 0.55% of participants with no VFL were recurrent fallers compared to 3.4% of participants with unilateral VFL (*P* < 0.05) or 3.4% of participants with bilateral VFL (*P* < 0005) (adjusted for age, sex, and moderate/severe disability). Association remained after adjustment for visual acuity

Lord and Dayhew, 2001 [13]	At visual tests: *n* = 156 Females: 63% Completed followup: *n* = 148	76.5 ± 5.1 63–90	Falls marked in a questionnaire by participants monthly	1 yr	Poor depth perception (RR = 2.26, 95% CI = 1.24–4.14), binocular poor low contrast visual acuity (RR = 2.08, 95% CI = 1.17–3.71), poor stereoacuity (RR = 1.99, 95% CI = 1.11–3.59), and poor distant-edge-contrast sensitivity (RR = 1.93, 95% CI = 1.01–3.68) risk factors for recurrent falls after adjustment for age, but poor visual acuity, reduced lower visual field size, and reduced near contrast sensitivity not risk factors.

Tromp et al., 2001 [5]	*n* = 1285 Females: 51%	75.2 ± 6.5 64.8–88.2	Falls reported weekly on a calendar posted every 3 mo Alternatively reported by telephone	1 yr	Subjective visual acuity impairment was a risk factor (OR = 2.6, 95% CI = 1.8–3.8) in unadjusted models.

Tromp et al., 1998 [14]	*n* = 1469 Females: 52%	72.6 ± 6.6 61.8–85.5	Questions about falls during the year before follow-up visit 3 yrs after baseline visit	1 yr	Subjective visual impairment a risk factor for recurrent falls (OR = 1.6, 95% CI = 1.1–2.3, unadjusted).

Luukinen et al., 1996 [15]	*n* = 788 Females: 63%	76.1 ± 4.9 70–92	Falling diary returned after each fall Participants not returning diary in 3 mo, were contacted by phone	2 yrs The population examined half-way through followup.	Self-reported ophthalmic disease a risk factor (RR = 1.5, 95% CI = 1.00–2.21) for at least 2 falls.

Nevitt et al., 1989 [16]	*n* = 325 females: 82%	60−	Weekly by postcards	1 yr	Decreased depth perception an independent predictor for ≥3 falls after adjustment (OR = 2.1, 95% CI = 1.1–4.2). Decreased visual acuity, visual field loss, or poor contrast sensitivity not associated with multiple falls.

Lord et al., 1994 [3]	*n* = 414 Females: 100% Follow-up data: *n* = 341	73.6 ± 6.3 65–99	Falls recorded on a posted questionnaire every 2 mo	1 yr	After controlling for age, there was a difference in low contrast visual acuity (*P* < 0.01) and contrast sensitivity (*P* < 0.01) between nonmultiple fallers and multiple fallers. High contrast visual acuity not a significant risk factor for falls.

Residents in intermediate care					
Clark et al., 1993 [17]	At baseline: *n* = 81 Females: 94% Followup data: *n* = 76	83.3 ± 5.8 70–97	Questionnaires about falls given monthly Nursing staff hold fall record book	1 yr	Visual field defects, cataract, retinopathy, or degeneration no risk factors for multiple. Impaired visual acuity more common in multiple fallers (RR = 1.79, 95% CI = 1.06–3.03, unadjusted).

Lord et al., 1991 [18]	*n* = 95 Females: 83% Completed follow-up: *n* = 84	82.7 ± 6.6 59–97	Falls recorded monthly with questionnaire and fall record book of staff.	1 yr	Multiple fallers had poorer contrast sensitivity (*P* < 0.01, adjusted for age) than nonmultiple fallers. No difference in best-corrected visual acuity after controlling for age between multiple fallers and nonmultiple fallers.

Tinetti et al., 1986 [19]	*n* = 79 Females: 68%	79 61–92	The staff reported falls to incident reports.	3 mo	Poor corrected distant vision in both eyes a risk factor for recurrent falling (RR = 3.5, *P* ≤ 0.05 unadjusted). Results about near vision not announced.

Graafmans et al., 1996 [20]	*n* = 354 Females: 85%	83 ± 6 70−	Falls recorded weekly on a calendar.	28 we	Self-reported distant vision loss not a risk factor for recurrent falls (OR = 1.7, 95% CI = 0.9–3.5), when adjusted for age and sex.

Institutionalized populations					
Luukinen et al., 1995 [21]	*n* = 93 Females: 76%	81.2 ± 5.8 70−	The staff reported falls by a postal diary after each fall. Medical records were checked.	2 yrs The population examined half-way through followup	An ophthalmic disease (asked by a postal questionnaire, nursing staff helped participants) an independent risk factor for recurrent falls (OR = 6.7, 95% Cl = 1.33–33.4) in a logistic regression analysis.

Retrospective studies					

Community-dwelling populations					
Ivers et al., 1998 [22]	Data about falls available: *n* = 3299 females: 57%	66.1 ± 9 49−	Participants were asked about all falls during the previous 12 mo.	1 yr retrospectively	Poor visual acuity wearing current glasses (prevalence ratio = 1.9, 95% CI = 1.2–3.0 after adjustment for confounders), poor contrast sensitivity (PR = 1.2, 95% CI = 1.1–1.3), and visual field loss (PR = 1.5, 95% CI = 1.0–2.3) associated with recurrent falls. Being unable to recognize a face across the street, see the TV, or read a newspaper were not significant risk factors after controlling for confounders. Posterior subcapsular cataract (PR = 2.1, 95% CI = 1.0–4.3) was associated with recurrent falls, but age-related macular degeneration, DM retinopathy, glaucoma, and cortical or nuclear cataract were not.

Rossat et al., 2010 [23]	*n* = 1066	65−	Participants were asked about all falls during the previous 12 mo.	1 yr retrospectively	Poor visual acuity was statistically significantly associated with recurrent falls (*P* = 0.006) after adjustment for potential confounders.

Van Nieuwenhuizen et al., 2010 [24]	*n* = 639 Females: 73%	78.5 ± 7.8	Participants were asked about all falls during the previous 12 mo.	1 yr retrospectively	Subjective impaired vision was not a risk factor for falling in a multivariate regression model.
